# Intrauterine growth restriction and hypospadias: is there a connection?

**DOI:** 10.1186/1687-9856-2014-20

**Published:** 2014-10-15

**Authors:** Min-Jye Chen, Charles G Macias, Sheila K Gunn, Jennifer E Dietrich, David R Roth, Bruce J Schlomer, Lefkothea P Karaviti

**Affiliations:** 1 Section of Pediatric Diabetes and Endocrinology, Department of Pediatrics, Baylor College of Medicine, Texas Children’s Hospital, Houston, TX 77030, USA; 2 Evidence-Based Outcomes Center and Center for Clinical Effectiveness, Baylor College of Medicine, Texas Children’s Hospital, Houston, TX 77030, USA; 3 Division of Pediatric and Adolescent Gynecology, Department of Obstetrics and Gynecology, Baylor College of Medicine, Texas Children’s Hospital, Houston, TX 77030, USA; 4 Division of Pediatric Urology, Department of Surgery, Baylor College of Medicine, Texas Children’s Hospital, Houston, TX 77030, USA; 5 Department of Urology, University of Texas Southwestern, Dallas, TX 75207, USA

**Keywords:** Intrauterine growth restriction, Fetal growth restriction, Small for gestational age, Low birth weight, Placental insufficiency, Hypospadias

## Abstract

Hypospadias is one of the most common congenital malformations of the genitourinary tract in males. It is an incomplete fusion of urethral folds early in fetal development and may be associated with other malformations of the genital tract. The etiology is poorly understood and may be hormonal, genetic, or environmental, but most often is idiopathic or multifactorial. Among many possible risk factors identified, of particular importance is low birth weight, which is defined in various ways in the literature. No mechanism has been identified for the association of low birth weight and hypospadias, but some authors propose placental insufficiency as a common inciting factor. Currently, there is no standardized approach for evaluating children with hypospadias in the setting of intrauterine growth restriction. We reviewed the available published literature on the association of hypospadias and growth restriction to determine whether it should be considered a separate entity within the category of disorders of sexual differentiation.

## Introduction

Hypospadias is a common congenital malformation in males, occurring in as many as 1 in 125 live male births, with some variation based on ethnicity [1-3]. Defined as an abnormal urethral opening on the ventral surface of the penis, it may be associated with other genitourinary anomalies such as cryptorchidism. Despite some argument to the contrary [3-7], many recent studies have documented an increase in the incidence of hypospadias, both in the United States [8,9] and worldwide [10-13].

Identifying the cause of hypospadias remains a challenge for pediatricians, endocrinologists, and urologists, as the etiology is varied and often idiopathic or multifactorial, particularly for isolated hypospadias. Some cases have a defect in hormonal synthesis, such as in 5-alpha reductase deficiency [14] or androgen insensitivity syndrome [15]. Genetic causes include certain syndromes (e.g., Smith-Lemli-Opitz syndrome [16] and others [17]), abnormalities in sex chromosomes, or mutations in specific genes involved in sexual differentiation [18-20]. Environmental causes also contribute to the development of hypospadias. For example, an increase in the use of pesticides and other endocrine-disrupting chemicals may contribute to the recent increase in incidence [21-25]. Among other maternal-fetal factors, low birth weight (LBW) has been associated with hypospadias, although the mechanism is unclear. Evaluation of this relationship is complicated by inconsistent definitions for LBW or intrauterine growth restriction (IUGR) in the literature. The purpose of this article is to review the association of LBW or IUGR with hypospadias and to determine if hypospadias and IUGR should be considered a distinct entity within disorders of sexual differentiation (DSD) that would require a different process of diagnostic evaluation and treatment.

## Methods

To elucidate the association between IUGR or LBW and hypospadias, we conducted a review of the available literature, using PubMed, Cochrane Library, and Google Scholar to answer the following questions:

1. Is growth restriction or LBW associated with hypospadias independently of other related factors, including gestational age?

2. In patients with IUGR and hypospadias, are the diagnostic evaluation and management of the hypospadias different from those for patients without IUGR?

We evaluated studies published in English, including case reports, observational studies, and controlled trials, that describe the relationship between hypospadias and LBW, IUGR, or small for gestational age. Search terms included hypospadias, fetal growth restriction, intrauterine growth restriction, small for gestational age, placental insufficiency, and low birth weight.

## Results

### Definitions

Descriptions of growth restriction vary in the literature, often with definitions based purely on birth weight, size for gestational age, or prenatal measurements. As a result, direct comparisons between studies are difficult. LBW and “small for gestational age (SGA) are both postnatal diagnoses. LBW refers strictly to birth weight [26], whereas SGA may refer to abnormal weight or length for gestational age [27,28]. In contrast, IUGR is primarily an obstetric diagnosis, as it depends on prenatal measurements. The term applies when the estimated fetal weight (EFW) is less than expected for gestational age, usually with the restriction persisting over some period of time, though a length of time requirement is not specified in most guidelines [29-31].

There is controversy over the definitions for SGA and IUGR. Though most authors use a cutoff of less than 10th percentile for gestational age for both SGA and IUGR, many researchers propose using more stringent definitions, as most infants who meet the 10th percentile criterion are constitutionally small and have normal perinatal outcomes [27,32,33].

Most studies evaluating the association of growth restriction with hypospadias use SGA or LBW definitions, sometimes interchangeably with IUGR. Although IUGR may lead to the diagnoses of SGA or LBW, the terms are not interchangeable. An infant may be born SGA without having had IUGR, or may have had a short period of IUGR without being SGA. Infants who are SGA may or may not be LBW, depending on gestational age. Pathologic growth restriction may be caused by genetic anomalies such as single gene mutations or chromosomal abnormalities, infections, placental disease, and maternal factors [34]. Studies have linked true fetal growth restriction to multiple congenital anomalies (including, but not limited to, hypospadias) [35-39], increased morbidity in the neonatal period [40], and long-term effects such as neurodevelopmental differences [41], short stature, increased risk of obesity, and metabolic syndrome [42]. Thus, identifying those infants at greatest risk of morbidity calls for a clear definition of IUGR.

### Is growth restriction or LBW associated with hypospadias independently of other related factors, including gestational age?

For decades, epidemiological studies (Table 1) have found that infants with hypospadias have birth weights lower than those of infants without hypospadias [2,35,36,43-46], although the difference in birth weights is not always statistically significant [47]. Because most studies evaluated multiple risk factors, the determination of whether LBW is independently associated with hypospadias or secondary to another risk factor such as maternal age or gestational age remains unknown. More recent population-based studies independently associated birth weight with hypospadias, even after accounting for possible confounders such as prematurity, multiple gestation, and use of assisted reproductive technologies [4,13,37,48,49].

**Table 1:** Summary of epidemiological studies associating hypospadias with low birth weight

**Study (year)**	**Dataset location (years)**	**Number of subjects with hypospadias**	**Definition**	**Findings**	**Other positive associations**
Chen (1971) [43]	Children’s Hospital of Michigan (1961–1967)	50	NA	Mean birth weight: hypospadias 2.7 kg, expected 3.3 kg	Parity
				p < 0.001	
Sweet (1974) [2]	Rochester, Minnesota, USA (1940–1970)	113	LBW: <2500 g	Presence of LBW in hypospadias 9%	
				Control 2%	
				No p value given	
Kallen (1982) [44]	Sweden (1965–1979)	1357	LBW: <2500 g	Presence of LBW in hypospadias 8.5%, Expected 4.2%	Prematurity
				p < 0.001	
Calzolari (1986) [45]	Emilia Romagna, Italy (1978–1983)	168	NA	Mean birth weight: hypospadias 2.97 kg, Controls 3.39 kg	Mother’s age at menarche, threatened abortion, use of progestins in pregnancy, gestational age
				p < 0.001	
Kallen (1986) [46]	Multiple: Denmark, Hungary, Italy, Mexico, South America, Spain, Sweden (years vary 1967–1982)	7491	LBW: <2500 g	Presence of hypospadias in LBW:	Maternal age, parity, gestational age, twin pregnancy
				RR 1.8-2.3 (varied by country)	
Khoury (1988) [35]	Atlanta, Georgia, USA (1975–1984)	1111	IUGR: <10 percentile birth weight for gestational age	Presence of IUGR in hypospadias:	Not evaluated
				RR (95% CI): 2.7 (2.3-3.1)	
Stoll (1990) [47]	Alsace, France (1979–1987)	176	NA	Mean birth weight: hypospadias 3.19 kg, controls 3.3 kg	Placental weight
				OR 2.05 (95% CI 0.73-5.74)	
Mili (1991) [36]	Atlanta, Georgia, USA (1978–1988)	919	NA	Presence of hypospadias in LBW:	Not evaluated
				Adjusted RR:	
				<1500 g: 3.3	
				1500-1999 g: 3.3	
				2000-2499 g: 2.2	
Riley (1998) [37]	Victoria, Australia (1983–1995)	2012	LBW <2500 g	Presence of LBW in hypospadias:	
				RR (95% CI): 2.23 (1.88-2.65)	
Akre (1999) [48]	Sweden (1983–1993)	1220	NA	Presence of LBW in hypospadias: Adjusted OR (95% CI):	Maternal age, parity, severe pre-eclampsia, other congenital malformations
				<1500 g: 6.02 (2.51-14.41)	
				1500-2500 g: 2.57 (1.71-3.85)	
Weidner (1999) [49]	Denmark (1983–1992)	1345	NA	Presence of LBW in hypospadias: Adjusted OR (95% CI):	Sibling with hypospadias, previous maternal history of stillbirth
				<2500 g: 3.42 (2.83-4.13)	
				2500-2599: 1.76 (1.47-2.10)	
Carmichael (2003) [4]	California, USA (1984–1997)	5838	NA	Presence of LBW in hypospadias:	White ethnicity, maternal education, maternal age, parity
				Adjusted RR (95% CI)	
				<1500 g: 2.46 (1.65-3.68) to 57.5 (31.8-104) depending on severity and other anomalies	
				1500-2499 g: 2.16 (1.73-2.69) to 18.8 (12.4-28.5)	
Carlson (2009) [58]	Nova Scotia, Canada (1980–2007)	995	NA	Birth weight in different severities of hypospadias:	Maternal age
				Adjusted OR 1.00, 95% CI 0.99-1.00	
Ghirri (2009) [59]	Italy (2001–2004)	234	SGA: <10 percentile for gestational age	Prevalence of hypospadias in SGA:	None
				5.28 per 1000 live births (compared to 2.56 per 1000 in AGA), p < 0.01	
				Significance only in moderate-severe hypospadias	
Nordenvall (2014) [13]	Sweden (1973–2009)	7974	SGA: <2 SD below mean	Presence of SGA in hypospadias:	Parental origin, maternal body mass index, in vitro fertilization, twin pregnancy
				Adjusted OR (95% CI):	
				4.15 (3.87-4.56)	

Case control and cohort studies also have found associations between hypospadias and birth weight. Birth weights were lower for boys with hypospadias than for those without hypospadias [50], and hypospadias also was more common in SGA or LBW infants [51]. These studies reported no relationship between gestational age and the frequency of hypospadias.

LBW also is associated with other genital anomalies, including cryptorchidism and more severe forms of DSD. A recent analysis of the International Disorders of Sex Development (I-DSD) registry found that as many as 23 percent of patients with male DSD also had SGA [52]. Patients with more severe anomalies, including hypospadias and undescended testes, have higher rates of IUGR than those with less severe anomalies, such as hypospadias and descended testes [53]. Birth weights and/or lengths are lower in patients with an unknown cause for DSD than in patients with identified causes for DSD, suggesting that growth retardation, particularly early in gestation, may be associated with abnormal testicular differentiation or DSD [54,55].

Twin studies support the relationship between restricted fetal growth and hypospadias by eliminating genetic and external environmental factors. In monozygotic twins discordant for hypospadias, the twin with the lower birth weight more commonly had hypospadias, and the difference in weight was significant [38,56,57]. This finding suggests that environmental factors specifically associated with the LBW twin, such as decreased placental blood supply, are involved in the development of hypospadias. Interestingly, one study did find a lower risk of hypospadias in twins compared to singletons when adjusted for weight, although this study did not compare weights between discordant twins [49].

Despite the evidence supporting the association between LBW and hypospadias, some debate continues. Determining causality is difficult due to the nature of the available studies. In addition, some studies find either no correlation between hypospadias and birth weight after accounting for confounders [58] or that birth weight was a risk factor only for severe hypospadias [59]. However, the populations in these studies were smaller than those in the studies that found a broader association between LBW and hypospadias.

### Possible mechanisms

Hypospadias is the result of incomplete fusion or failure of fusion of the urethral folds during early fetal development. Two basic phases occur in the development of a male phenotype. The first phase is testicular development, which typically is determined by the presence of the Y chromosome, specifically the SRY gene, although many other genes also participate in testicular development. The second phase involves androgen effects through production by the testes as well as downstream responses. Defects in either phase may lead to abnormal sexual differentiation [60]. Typically, the external genitalia are undifferentiated until approximately week 8 of gestation, at which time differentiation to male external genitalia begins. During this critical period, human chorionic gonadotropin (HCG) induces masculinization by stimulating the production of testosterone and dihydrotestosterone (DHT) by the interstitial cells of the fetal testes. Fusion of the urethral folds usually is complete by approximately week 16 of gestation; thus, environmental or hormonal disturbances, including any underlying causes of IUGR, must occur before this time to cause hypospadias [61]. Although establishing conclusively that early IUGR is associated with development of hypospadias is difficult, one retrospective cohort study did find a higher rate of hypospadias in infants who were SGA in all three birth measures (i.e., weight, length, and head circumference), compared to those considered appropriate or large for gestational age. The combination of SGA measurements is suggestive of growth restriction early in gestation [51].

Several studies have associated birth weight and hypospadias to disturbances in the fetal-placental-maternal unit. In SGA infants, including those with hypospadias, some researchers have noted an association with maternal hypertension, oligohydramnios, and preterm birth [62,63]. Placental and fetal weight tend to be lower in hypospadic infants, independent of gestational age [47,62,64,65], and the severity of hypospadias is increased in SGA infants [63].

Other studies looking more directly at the placenta and fetal growth suggested placental insufficiency as an inciting factor for both LBW and hypospadias. Histopathologic examination of placentas of patients with hypospadias and LBW revealed abnormalities such as low placental weight, evidence of infarction, calcifications, abnormal cord insertion, and other degenerative changes [57,64].

Based on the associations between LBW or IUGR and hypospadias, some researchers have hypothesized that placental insufficiency in the first trimester may cause inadequate HCG delivery to the fetus, with the resultant fetal production of testosterone and DHT being inadequate to induce complete virilization [51,56,61]. The timing is critical, as later placental insufficiency might cause IUGR but not hypospadias, as the fusion of the urethral folds is complete by week 16. This hypothesis has been challenged, as some studies have found no difference in maternal HCG levels before 18 weeks gestation in patients with hypospadias compared to controls [66] and higher than normal second-trimester maternal HCG levels in mothers with placental dysfunction [67]. However, these studies did not necessarily measure maternal HCG levels in the period of time when urethral fusion would be expected to occur. Regardless, normal or high maternal HCG levels do not guarantee that sufficient levels are available to the fetus to produce testosterone and DHT levels adequate for virilization, as the HCG levels seen by the fetus is dependent on an intact placental vessel delivery system.

### In patients with IUGR and hypospadias, are the diagnostic evaluation and management of the hypospadias different from those for patients without IUGR?

**Figure 1:**
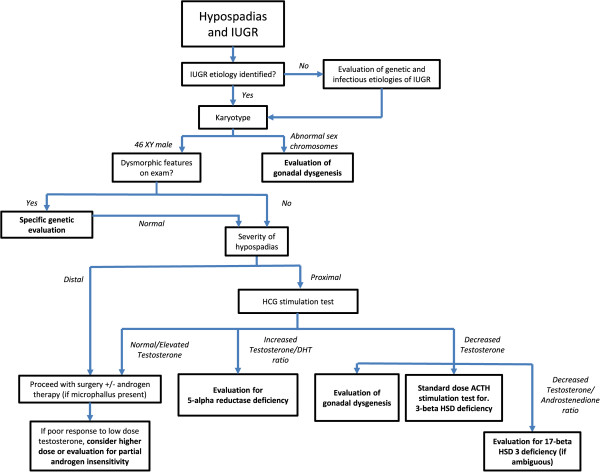
Proposed diagnostic algorithm for initial hormonal and genetic testing for etiology of hypospadias in setting of IUGR.

We did not identify studies that discussed the diagnostic evaluation and management of hypospadias specifically in the setting of IUGR or LBW. Although some genetic syndromes feature both IUGR and hypospadias (e.g., Wolf-Hirschhorn (4p-) syndrome [68] and others), they are rare, and the evidence is insufficient to suggest that the evaluation of hypospadias should differ significantly in IUGR patients compared to normal-weight patients. Evaluation of patients with both IUGR and hypospadias should include a systematic, evidence-based approach, such as the algorithm provided in Figure 1, summarized below.

For infants with hypospadias and evidence of IUGR, assessment should begin with evaluation of an underlying etiology for the growth restriction, including genetic abnormalities or infection [34], if a cause of poor growth was not identified prenatally. Karyotype or chromosomal microarray evaluation would be particularly useful to identify genetic etiologies of severe hypospadias as well as IUGR, as patients with sex chromosome abnormalities, including but not limited to 46 XY/45 XO or 46 XX with virilization, can present with some degree of DSD [69]. If sex chromosome aneuploidy is present, one should consider further evaluation for gonadal dysgenesis.

**Figure 2:**
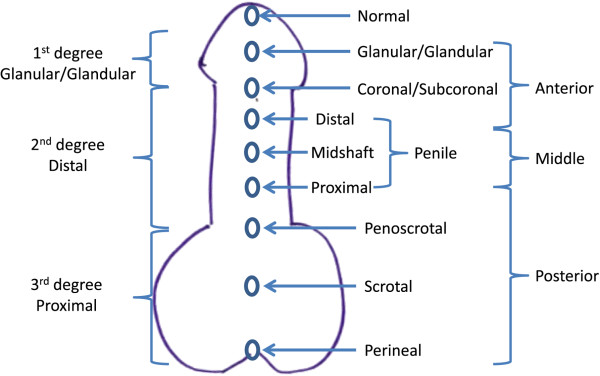
**Diagram of commonly used classifications of hypospadias, based on location of urethral meatus.** These categories were described by Boisen [70], Duckett [71], Hadidi [72], and Smith [73].

In the evaluation of hypospadias in infants known to be 46 XY, the severity of hypospadias must be considered, as pathology identified is more often in severe cases. The classification of hypospadias usually depends on the location of the urethral opening (Figure 2) [70-73], as well as the presence of other genitourinary anomalies such as cryptorchidism. Some studies have evaluated the hormonal status in patients with hypospadias, with inconsistent protocols and results. However, evaluation of HCG-stimulated testosterone and DHT production may be the most useful in diagnosing an endocrine etiology for hypospadias. Patients with hypospadias may have abnormal stimulated production of testosterone, especially if other genitourinary abnormalities are present on exam [74-76]. Increased testosterone-to-DHT ratio suggests 5-alpha reductase deficiency, whereas decreased testosterone production may indicate testicular dysgenesis or defects in steroidogenesis, including 3-beta hydroxysteroid dehydrogenase deficiency or 17-beta hydroxysteroid deficiency. Elevated androgen levels may occur in partial androgen insensitivity. If dysmorphic features or multiple congenital anomalies exist, further genetic evaluation may be indicated for evaluation of known syndromes.

Surgery is the definitive treatment for hypospadias, and preoperative androgen therapy often helps facilitate the repair, particularly in patients with small penile size, although its use remains controversial [77]. Preoperative androgen therapy increases penile length, diameter, or circumference with minimal and transient side effects [78-83]. Intramuscular testosterone is the preferred therapy [77,79-81], although some studies have reported positive outcomes with topical testosterone and DHT [78,83,84]. The dosages of intramuscular testosterone varied, but studies using low-dose testosterone enanthate (2 mg/kg or 25 mg) or equivalent doses of other formulations had beneficial results in penile size similar to those of studies using higher doses. For patients with 5-alpha reductase deficiency or partial androgen insensitivity, a higher dose or multiple courses of testosterone therapy may be needed [85]. DHT, if available, may also be useful in patients with 5-alpha reductase deficiency [86].

## Conclusions

Hypospadias is one of the most common congenital malformations in males, but its etiology remains poorly understood. LBW or growth restriction, which often is associated with hypospadias, is one of the risk factors that researchers have evaluated. The mechanism is unknown, but placental insufficiency as a possible cause of both IUGR/LBW and hypospadias should be studied further. The current evidence is insufficient to recommend that patients with IUGR and hypospadias be assessed and managed differently from patients of normal weight. Further studies are needed to develop a standardized algorithm for diagnostic evaluation and management to minimize cost and patient discomfort and to determine whether hypospadias in the setting of IUGR should be considered a separate DSD entity in the future.

## Abbreviations

IUGR: Intrauterine growth restriction; LBW: Low birth weight; SGA: Small for gestational age; EFW: Estimated fetal weight; HCG: Human chorionic gonadotropin; DHT: Dihydrotestosterone; DSD: Disorders of sex development; OR: Odds ratio; RR: Relative risk; CI: Confidence interval.

## Competing interests

The authors declare that they have no competing interests.

## Authors’ contributions

MJC performed the literature review and drafted the manuscript. CM provided the training necessary for composing an evidence-based review article, critically appraised the paper, and provided key changes to the intellectual content. JD, SG, DR, and BS also critically reviewed the manuscript and made key changes with respect to the design and intellectual content. LK was involved in the initial conception and design of the manuscript as well as critical review and key changes to the intellectual content. All authors read and approved the final manuscript.

## Authors’ information

MJC is a third-year clinical Pediatric Endocrinology fellow at Baylor College of Medicine, Texas Children’s Hospital.

CM is an Associate Professor in the Department of Pediatrics, Section of Emergency Medicine at Baylor College of Medicine, Texas Children’s Hospital. He is also the director for the Evidence-based Outcomes Center and Center for Clinical Effectiveness at Texas Children’s Hospital.

JD is an Associate Professor, Section Chief, and Fellowship Director of Pediatric & Adolescent Gynecology at Baylor College of Medicine, Texas Children’s Hospital.

SG is an Associate Professor in the Department of Pediatrics, Section of Pediatric Diabetes and Endocrinology, at Baylor College of Medicine, Texas Children’s Hospital.

DR is a Professor of Urology, Pediatrics, and Obstetrics/Gynecology, and Chief of Pediatric Urology at Baylor College of Medicine, Texas Children’s Hospital.

BS is an Assistant Professor of Pediatric Urology at University of Texas Southwestern.

LK is a Professor in the Department of Pediatrics, Section of Pediatric Diabetes and Endocrinology, at Baylor College of Medicine, Texas Children’s Hospital.
